# Quantitative bias analysis for unmeasured confounding in unanchored population-adjusted indirect comparisons

**DOI:** 10.1017/rsm.2025.13

**Published:** 2025-03-24

**Authors:** Shijie Ren, Sa Ren, Nicky J. Welton, Mark Strong

**Affiliations:** 1 School of Medicine and Population Health, University of Sheffield, Sheffield, UK; 2 Population Health Sciences, Bristol Medical School, University of Bristol, Bristol, UK

**Keywords:** quantitative bias analysis, unmeasured confounding, unanchored simulated treatment comparison, population-adjustment, indirect treatment comparison

## Abstract

Unanchored population-adjusted indirect comparisons (PAICs) such as matching-adjusted indirect comparison (MAIC) and simulated treatment comparison (STC) attracted a significant attention in the health technology assessment field in recent years. These methods allow for indirect comparisons by balancing different patient characteristics in single-arm studies in the case where individual patient-level data are only available for one study. However, the validity of findings from unanchored MAIC/STC analyses is frequently questioned by decision makers, due to the assumption that all potential prognostic factors and effect modifiers are accounted for. Addressing this critical concern, we introduce a sensitivity analysis algorithm for unanchored PAICs by extending quantitative bias analysis techniques traditionally used in epidemiology. Our proposed sensitivity analysis involves simulating important covariates that were not reported by the comparator study when conducting unanchored STC and enables the formal evaluating of the impact of unmeasured confounding in a quantitative manner without additional assumptions. We demonstrate the practical application of this method through a real-world case study of metastatic colorectal cancer, highlighting its utility in enhancing the robustness and credibility of unanchored PAIC results. Our findings emphasise the necessity of formal quantitative sensitivity analysis in interpreting unanchored PAIC results, as it quantifies the robustness of conclusions regarding potential unmeasured confounders and supports more robust, reliable, and informative decision-making in healthcare.

## Highlights

### What is already known

- Population-adjusted indirect comparisons (PAICs) are increasingly used to account for differences in patient characteristics between two treatments evaluated in different studies.
- Unanchored PAICs such as matching-adjusted indirect comparison and simulated treatment comparison, are commonly used when the evidence involves single-arm trials.
- Confounding significantly challenges the reliability of using evidence from single-arm trials to inform the relative effect of treatments.

### What is new

- We extended quantitative bias analysis for unmeasured confounding to unanchored PAICs, presenting a set of graphical tools to explore the sensitivity of indirect treatment effect estimates to the presence of unmeasured confounder(s).
- We illustrated how our proposed approach can quantify the potential bias associated with unmeasured confounding where certain prognostic factors and/or effect modifiers are not reported in the comparator study, aiding decision-making via a real-world case study.
- Our findings emphasise the necessity of formal quantitative sensitivity analysis in the interpretation of unanchored PAIC results, ensuring more robust, reliable, and informative decision-making in healthcare.

### Potential impact for Research Synthesis Methods readers

- Sensitivity analysis can quantify how robust the conclusion is to the potential unmeasured confounder(s) and should be used to aid decision-making.
- The quantitative bias analysis approach may have applicability in other areas where decision-making relies on untestable assumptions.

## 1 Introduction

Health technology assessment (HTA) evaluates the clinical and economic impacts of health technologies. It provides systematic evidence to policymakers and healthcare providers with the goal to ensure healthcare meets high-quality standards and offers optimal value for money. The evidence that informs HTA decision-making encompasses a wide variety of types with well-conducted randomised controlled trials (RCTs) regarded as the gold standard for evaluating relative treatment effects.^1^ However, an RCT may not be feasible due to ethical and practical challenges, for example, in rare diseases and highly targeted patient populations. In these cases, ‘single-arm trials’ in which all patients receive the same treatment may be conducted. A review of submissions to HTA bodies revealed a significant year-on-year increase in the submissions with single-arm trials (from 8 in 2011 to 102 in 2019).^2^ Lack of randomisation of single-arm trials raises a particular challenge in HTA where the estimate of the relative treatment effect against an intervention used in routine practice is required for decision-making.

Unanchored indirect treatment comparison is required to estimate the relative treatment effect based on single-arm trials without a common comparator. In HTA, it is common that the evidence for the efficacy of the comparator treatment is available only as a set of published summary statistics (i.e., as aggregate data), and that the individual patient-level data (IPD) from the single-arm trial of the treatment of interest are available to the pharmaceutical company that developed the treatment and is conducting the analysis. This is the setting that we are considering in this article.

Due to the inherent lack of randomisation in single-arm trial designs, confounding issues significantly challenge the reliability of using evidence from these studies to inform the relative effect of treatments. Population-adjusted indirect comparison (PAIC) methods such as the matching-adjusted indirect comparison (MAIC)^3^^,^ ^4^ and the simulated treatment comparison (STC)^4^^,^ ^5^ were developed with an aim to adjust for differences in baseline characteristics between the two study population in the case IPD are only available for one of the studies. MAIC is based on propensity score weighting and STC is based on regression adjustment. Both methods provide an estimate of the relative treatment effect in the comparator study population where only aggregate data are available.

A valid unanchored MAIC/STC requires that all potential prognostic factors and effect modifiers are included in the analysis.^6^ Both approaches require sufficient overlap between the study populations. The STC approach also requires the correct specification of the form of the outcome regression model in order to provide unbiased estimates. The assumption of no unmeasured confounding is largely considered impossible to meet in an unanchored comparison,^6^^–^ ^8^ and all the previous simulation studies show the importance of adjusting for all relevant covariates in MAIC and STC to avoid bias.^9^^–^ ^14^ However, the selection of covariates is often restricted by the availability of the data. Information on the baseline characteristics for the comparator study population is often reported only for a smaller set of covariates compared to those that were measured in the company’s study. Adjustment using MAIC and STC would be limited only to the covariates reported in the comparator study, which is likely to lead to bias. There is currently no standard approach to quantifying this potential bias, which has led to caution in the use of results from unanchored MAIC/STC analyses in reimbursement decisions.

A recent review on the use of PAICs in practice show that there is a substantial increase in the use of these methods in recent years, with about three-quarters being unanchored and MAICs were applied significantly more frequently than STCs.^15^ Methodological review of these methods also highlighted that there are currently no methods to assess the robustness of PAIC with regard to the no unmeasured confounding assumption; and there is a pressing need for additional guidelines and recommendations regarding methodological and reporting standards to enhance the quality of such analyses in the future.^15^^,^ ^16^

In this article, we aim to address the limitation of the current unanchored PAIC methods where certain prognostic factor and/or effect modifiers are not reported in the comparator study. We firsty explain the impact of uncontrolled confounding in a simple case. We then introduce the concept of quantitative bias analysis (QBA) for unmeasured confounding in a general setting. We extend the QBA technique for uncontrolled confounding to unanchored PAIC methods. We present how sensitivity analysis could be conducted for important covariates that were not reported by the comparator study when conducting unanchored STC. We demonstrate the method through a real-world case study in metastatic colorectal cancer.

## 2 Consequences of uncontrolled confounding

We now demonstrate the exact bias associated with omitting a single binary unmeasured confounding variable 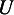 . Suppose that we know the full linear regression model to estimate the effect of treatment 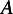 on an outcome 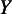 by controlling for a set of covariates 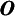 and 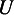 is (1)

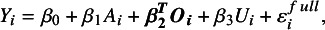

where 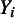 is the outcome of interest for individual 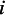 ; 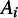 is the treatment variable for individual 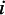 ; 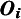 is a 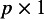 vector of observed 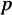 covariates for individual 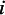 ; 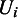 is the unmeasured covariate for individual 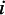 ; 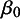 is the intercept; 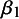 is the coefficient for the treatment variable; 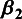 is a 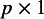 vector of coefficients for observed 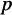 covariates; 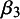 is the coefficient for the unmeasured covariate; and 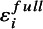 is the random error of the full model.

Because 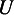 is unmeasured, we have to estimate the treatment effect using a restricted model without the covariate 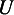 :(2)

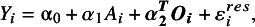

where 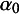 is the intercept; 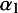 is the coefficient for the treatment variable; 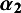 is a 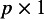 vector of coefficients for observed 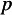 covariates; and 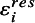 is the random error of the restricted model.

Cinelli and Hazlett (2020) showed that by applying the Frisch–Waugh–Lowvell (FWL) theorem,^17^ there is a closed form solution for the bias associated with the estimate of 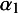 from the restricted model comparing with the estimate of 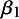 from the full model.^18^ The bias by omitting the unmeasured confounding variable 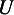 when estimating the treatment effect is (3)

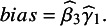




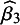
 is the least square estimate of the coefficient for the unmeasured 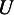 in the full model and it describes how the change in the values of the unmeasured confounding variable would impact the prediction of the outcome 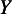 . That is, 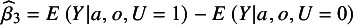 . 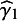 is the estimate of the coefficient of the regression of 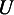 on the treatment 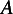 . It describes the difference in the predicted value of the unmeasured confounder 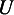 , when comparing individuals with the same covariates 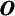 but different treatment 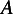 . That is, 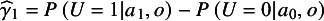 , where 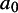 and 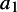 are the two possible treatments; 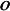 are the observed covariates.

Mathematically, the calculation of the bias for omitting multiple unmeasured covariates extends naturally. VanderWeele and Arch (2011) used the causal inference counterfactual framework to derive a general class of bias formulas for sensitivity analysis for causal effects when considering unmeasured confounding variables.^19^ The bias formulas generalise many of the existing sensitivity analysis results in the bias-modelling literature. The formulas were also very flexible in the sense that it does not presuppose a particular functional form relating the outcome and the observed covariates and treatment. VanderWeele and Arch proved that under some stronger assumptions their proposed bias formulas can be reduced to the product of 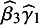 (Equation (3)) when 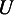 is binary in their analysis.^19^

## 3 Quantitative bias analysis (QBA) for unmeasured confounding

QBA is an umbrella term for the methods used to model systematic errors that may distort the results. This approach has a long history of development in the field of epidemiology, linking back to the study published by Cornfield et al. (1959).^20^ investigating the causal effect between smoking and lung cancer. The aim of the QBA is to quantitatively measure the direction, magnitude, and uncertainty associated with systematic errors on study results. The analyses can be broadly categorised to assess the impact of violations to the following assumptions^21^^,^ ^22^:

- no unmeasured confounders;
- selection, participation, and missing data are random within levels of adjusted covariates;
- no measurement error (including misclassification).

Details of the implementation of the QBA methods can be found in Fox et al. (2022) and good practices for applying QBA to epidemiological data can be found in Lash et al. (2014).^23^^,^ ^24^ In this article, we are only interested in the QBA methods used for unmeasured confounding. Leahy et al. (2022) summarised the methods available in this situation and grouped the methods based on the analytic approaches used.^25^ The basic idea behind the QBA methods is described below following Kawabata et al. (2023).^26^

A QBA requires a model (also known as a bias model) for the observed data (an outcome 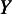 , an exposure/treatment 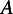 , and observed covariates 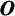 ) and unmeasured covariates 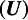 . The bias model includes one or more sensitivity/bias parameters, where the values of these parameters cannot be estimated from the data alone as it involves analysing unmeasured data 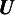 . An example of the sensitivity parameters is the strength of the association between unmeasured covariates and an exposure given measured covariates. When conducting the QBA, these sensitivity parameters need to be pre-specified, which leads to either deterministic or probabilistic QBA. In a deterministic QBA, some fixed values for the sensitivity parameters are specified and used in the analysis to obtain a range of results showing the impact of changing the sensitivity parameters. In a probabilistic QBA, a probability distribution for the sensitivity parameters would be specified and the results is obtained by average over this distribution to take into account uncertainty in the sensitivity parameters. However, this relies on the distribution being correctly specified, which is often challenging to achieve. Finally, a QBA could also be performed as a tipping point analysis with an aim to identify the values for the sensitivity parameters that would change the study conclusion. This is a similar approach to the threshold analysis in health economics, which answers the question ‘to what extent would the evidence need to change to alter the recommendation’.^27^

## 4 Sensitivity analysis for population-adjusted indirect comparisons (PAICs)

One of the major criticisms of unanchored MAIC and STC approach is that it relies on the strong assumption that both potential prognostic factors and effect modifiers are adjusted for. In practice, what could be adjusted for in the analysis depends on what were reported as baseline characteristics in both the company’s study and the comparator study. It is often that this information is limited in the comparator study and does not include all the important variables, which should be adjusted for.

We propose to apply QBA for PAICs where we only have IPD for one of the group and aggregate data for the other group. In theory, the general class of bias formulas proposed for mean difference, risk ratio and odds ratio by VanderWeele and Arch (2011)^19^ can be applied directly to the estimate from MAIC/STC as a sensitivity analysis for unmeasured confounding. This is because the general bias formulae do not depend on the method use for obtaining the estimate (a method of moments for MAIC or maximum likelihood method for STC), are applicable regardless of how the adjusted estimates are obtained (MAIC/STC); and also no assumptions are pre-specified for the interactions between treatment and covariates. However, as the dimension of the unmeasured confounders increases the implementation of the general bias formulas becomes more complex and less feasible due to the need to obtain multiple summations, integrations or a combination of both.

Below we describe an alternative sensitivity analysis approach for unmeasured confounding based on simulating potential confounder(s) for PAICs. Our approach allows for sensitivity analysis for important covariate(s) that were not reported by the comparator study when conducting unanchored STC. No additional assumptions are required for our approach. Moreover, our approach is easy to implement regardless of the dimension and data type of unmeasured confounders.

### 4.1 Principle of the QBA for PAICs

Let us assume the company’s study (Study B) has IPD available for the outcome of interest 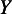 and 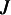 covariates 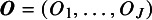 as well as 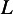 covariates 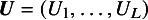 ; the comparator study (Study A) report the treatment effect for patients receiving treatment A, 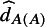 , and the summary statistics in terms of marginal mean [ 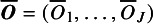 ] for 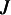 observed covariates 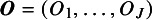 . The summary statistics [ 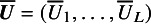 ] for 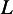 covariates 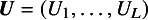 are not reported for Study A. Note the description of the method below refers to multiple unmeasured covariates. For a single unmeasured covariate, 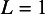 .

**Figure 1:**
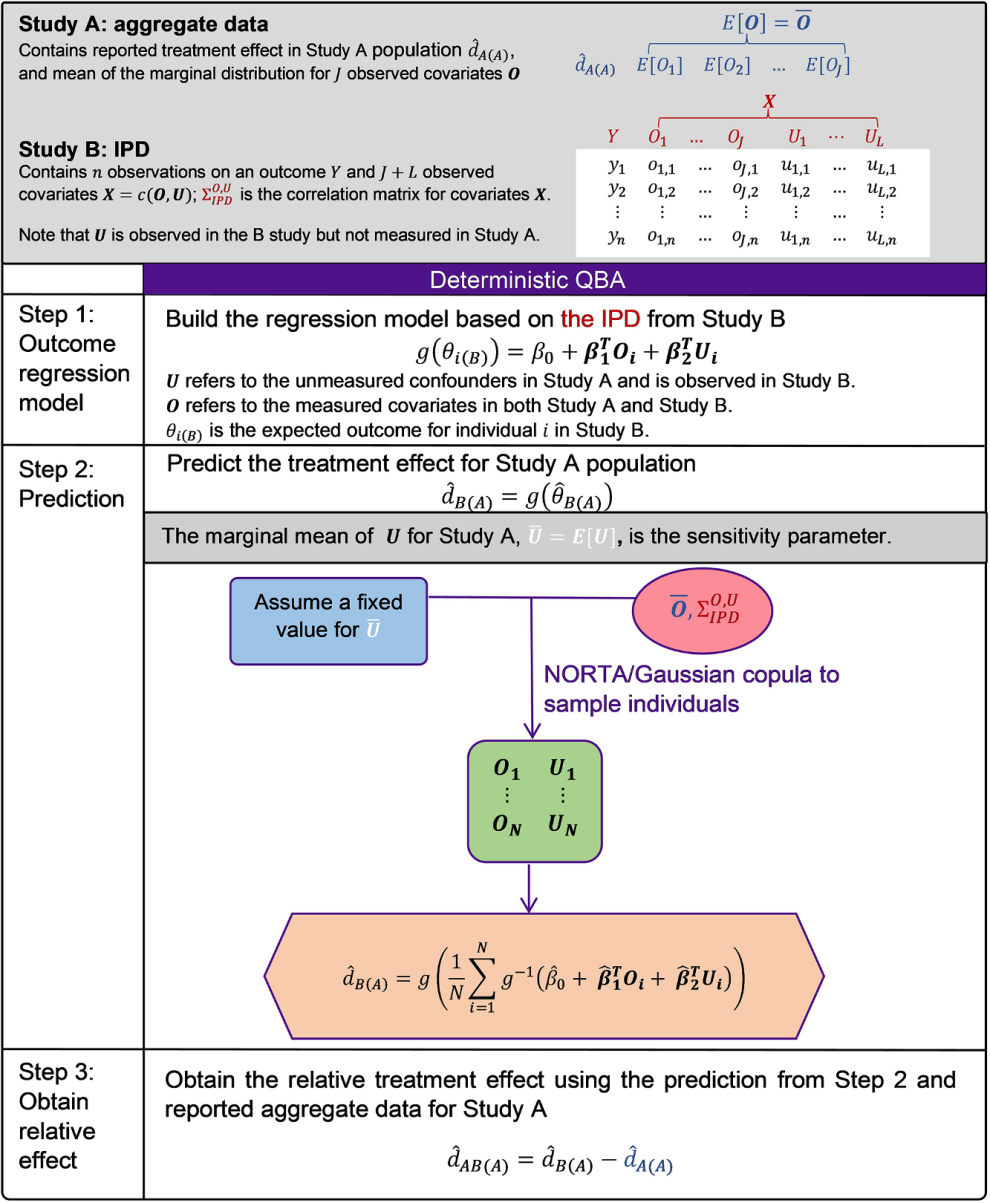
Illustration of the sensitivity analysis for unmeasured confounding for the unanchored STC analysis.

In a standard STC or MAIC approach, we can only adjust for the differences in the observed covariates 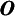 between Study A and Study B, and confounding bias will be present unless unmeasured covariates 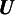 are balanced between the populations in the two studies. We can treat [ 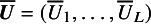 ] in Study A as sensitivity parameters and conduct QBA to assess the impact on not controlling 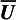 in the analysis. A graphical illustration of the available data from each study is presented in Figure 1.

QBA is technically feasible for both MAIC and STC. However, the sensitivity analysis should assess the impact of the full spectrum of possible values for 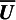 and this would include the cases where there would be very limited overlap on 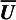 between the two studies. MAIC approach would suffer an extreme reduction of the effect sample size and may even fail to produce feasible weights. Hence, we will only present the QBA for the STC approach. A flow diagram and graphical illustration of the method is presented in Figure 1.

Ren et al. (2024) developed a novel way to implement unanchored STC by incorporating marginalisation and the NORmal To Anything (NORTA) algorithm (also is known as a Gaussian copula method) for sampling covariates, and demonstrated that the proposed approach is asymptotically unbiased in a simulation study.^28^ Our proposed QBA will follow this suggested implementation for unanchored STC. We briefly describe the unanchored STC approach here.

We assume that we have IPD from a single-arm study for treatment B (Study B) and aggregate data from another single-arm study for treatment A (Study A), and we are interested in estimating the relative treatment effect of treatment B versus treatment A using an unanchored STC analysis. STC is an outcome regression-based approach and has three main steps.

1. Build the regression model based on the IPD from Study B: 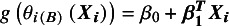 .
2. Predict the treatment effect for the Study A population via marginalisation/standardisation: 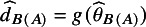 .
3. Obtain the relative treatment effect using the prediction from Step 2 and reported aggregate data for Study A: 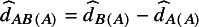 .

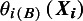
 is the expected outcome for individual 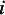 with covariate values 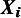 in Study B (e.g., the probability for binary outcomes); the subscript (*B*) indicates the population; 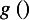 is an appropriate link function (e.g., the logit function for binary outcomes); 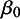 is the intercept; 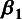 is a vector of coefficients for prognostic factors and effect modifiers; and 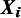 is the full covariate vector including prognostic factors and effect modifiers for individual 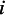 .


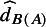
 is the predicted average effect of treatment B in the Study A population and is obtained by marginalisation/standardisation of the predicted conditional estimates for the sampled individuals in Study A; 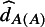 is the reported treatment effect of treatment A in the Study A population; and 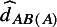 is the estimated relative treatment effect of B versus A in the Study A population.

When performing QBA for sensitivity analysis, we distinguish observed covariates 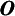 and unmeasured covariates 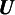 for the notation used in the regression models (Step 1). The equation in Step 1 becomes (4)

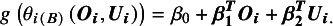



The marginal mean of 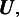 ( 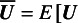 ]) used in Step 2 (predicting the treatment effect in the Study A population) are treated as sensitivity parameters.

### 4.2 Sensitivity analysis algorithm

In QBA, we assume fixed values for 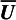 . For linear regression models with an identity link function for continuous outcome, 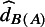 can be obtained using the ‘plugging-in’ mean covariates approach because of the linear relationship between the outcome and the predictors. The prediction can be obtained by plugging in the reported mean values for 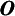 , 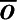 and fixed values for 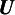 , 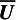 . The estimate of the treatment effect of B versus A in the comparator trial population is given by (5)

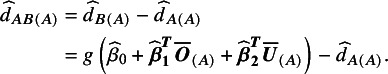



For regression models with non-identity link function, the ‘plugging-in’ approach would lead to aggregation bias.^29^^,^ ^30^ To deal with non-linearity, the adjusted absolute effect can be obtained by averaging the predictions of the sampled individuals. These covariates can be sampled using the NORTA algorithm based on the reported summary statistics for observed covariates from Study A, the assumed fixed values for the marginal mean of the unmeasured covariates, and the correlation structure from Study B (the study with IPD).^28^ The NORTA algorithm is summarised in Appendix 1 of the Supplementary Material.

We now describe the prediction procedure step-by-step in the unanchored STC using a binary outcome as an example. Let us assume 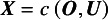 is the full covariate vector. For a binary outcome 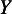 , (6)

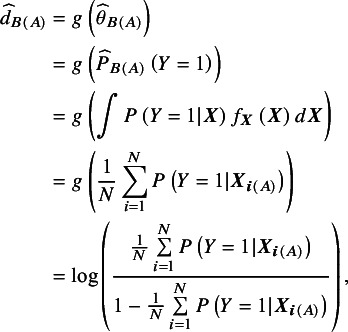

where 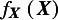 is the joint probability density function for 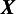 representing the Study A population if 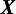 contains all continuous covariates; 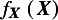 is the joint probability mass function if 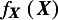 contains all discrete covariates; and is a joint density function with respect to an appropriate dominating measure if 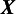 is a mixture of continuous and discrete covariates. Note that the integral is estimated using Monte Carlo.^31^

In the deterministic QBA, 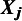 are random samples from 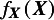 using the NORTA algorithm based on the marginal distribution of 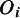 and 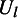 and the correlation structure from Study B, where the input for the marginal distribution of 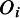 is based on the reported mean for 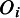 ( 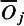 ) from Study A and the input for the marginal distribution of 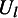 is based on the assumed fixed values for the mean of 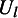 ( 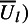 .

The general formula for the estimator 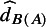 is(7)

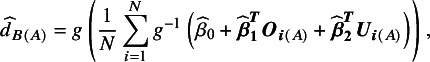

where we firstly obtain the predicted outcome on the natural scale for all 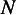 simulation samples for covariates 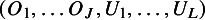 sampled from the joint covariate distribution, 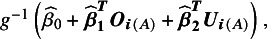 (e.g., the probability for binary outcomes); then we obtain the average predicted outcome on the natural scale (e.g., the average probability for binary outcomes); finally we transform the predicted outcome to the linear predictor scale using 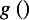 (e.g., the log odds for binary outcomes).

The estimate of the treatment effect of B versus A in the Study A population is given by (8)

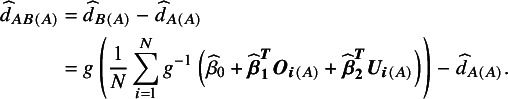



Because of the lack of a closed-form expression for this variance, the variance of 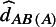 is computed using the non-parametric bootstrap method.^32^

This deterministic QBA allows us to perform sensitivity analysis to quantify the impact of the unmeasured prognostic variables and/or effect modifiers on the estimated treatment effect by varying the fixed values for 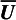 . It provides an assessment of the robustness of the STC results to assumptions about the unmeasured confounders. However, it does not capture the uncertainty in the marginal mean for the unmeasured confounders.

A probabilistic QBA can be conducted if a distribution for the marginal means for unmeasured confounders 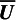 could be specified. However, obtaining this distribution in practice may be challenging, and it may require to perform a structured expert elicitation exercise^33^ or analysing the relationship between the observed and unmeasured covariates based on external data where data are available for all covariates and predicting the distribution for the marginal mean for the unmeasured confounders. We provide some initial suggestions in the discussion section.

## 5 Example: Metastatic colorectal cancer

We re-analysed the data from a randomised Phase III trial of panitumumab with infusional fluorouracil, leucovorin and oxaliplatin (FOLFOX4) versus FOLFOX4 alone as first-line treatment in patients with previously untreated metastatic colorectal cancer (the PRIME study: NCT00364013)^34^ to demonstrate the application of both deterministic and probability QBA for population adjustment methods to a real-world example. We obtained the IPD for this RCT from the Project Data Sphere® platform.^35^ The anonymous patient-level data contains 79% of the subjects randomly selected from the original trial dataset in each arm. In this example, we re-analysed one of the secondary outcome measures: objective response. We dropped the control arm (FOLFOX4) and treated the data in the intervention arm (panitumumab with FOLFOX4) as if the data were from a single-arm trial. We obtained summary statistics for the FOLFOX4 arm from an external source and applied the unanchored STC and deterministic sensitivity analysis to explore the impact of unmeasured confounding.

To illustrate the methods, we identified a study with aggregate data for the comparator FOLFOX4, which differed from the PRIME study in patient characteristics. Because the purpose of this example is to demonstrate the use of the QBA approach for PAICs, not to guide selection of the most appropriate studies for the comparator using external sources, we did not conduct a systematic literature review to obtain the summary data reported for the FOLFOX4 arm externally. Instead, Google scholar was used to perform the search using key words ‘FOLFOX4’ and ‘metastatic colorectal cancer’. We use the following criteria to select an appropriate study for this analysis: (i) the patient population was previously untreated metastatic colorectal cancer, (ii) a sufficient large number of baseline characteristics have been reported, and (iii) the reported baseline characteristics are not too similar to the PRIME study. There was no restriction on the type of study.

The most appropriate study that we identified was Cunningham et al. (2009)^36^ This study is a Phase III RCT comparing two different first-line 5-fluorouracil (5-FU) regimens with or without oxaliplatin in patients with previously untreated metastatic colorectal cancer. The treatment arm suitable to be used in our analysis is the oxaliplatin, 5-FU, and leucovorin (LV) arm, because continuous intravenous infusion (CIV) of 5-FU without LV was a common regimen used in the UK at the time this trial was conducted. The oxaliplatin, 5-FU and LV arm is divided into two subgroups: oxaliplatin plus 5-FU CIV group (*n* = 58) and FOLFOX4 group (*n* = 304). However, data are not available for the FOLFOX4 arm alone. The baseline characteristics and objective response rate are reported for the combined oxaliplatin, 5-FU and LV arm. In this example, we assume the treatment of oxaliplatin plus 5FU CIV is the same as FOLFOX4. Because the sample size in the oxaliplatin plus 5-FU CIV arm is relatively small compared to the FOLFOX4 arm (58 vs. 304), we do not anticipate a large bias associated with this assumption. We call the oxaliplatin, 5-FU, and LV arm in Cunningham et al.,^36^ the FOLFOX4 arm for the rest of the analysis.

The commonly reported clinical baseline characteristics and objective response data from the PRIME study and the FOLFOX4 arm from Cunningham et al. (2009)^36^ are presented in Table 1. There are some imbalances in the baseline characteristics between the panitumumab with FOLFOX4 arm in the PRIME study and the FOLFOX4 arm in Cunningham et al.^36^ The panitumumab + FOLFOX4 arm in the PRIME study has more patients younger than 65 years old, and slightly more patients with ECOG status 0/1, which indicate potentially heathier cohort than patients in the FOLFOX4 arm in Cunningham et al.^36^ However, it also has more patients with colon as the primary tumour site, more patients with more than two metastatic sites, less patients had prior adjuvant chemotherapy and more patients had prior surgery, which indicate potential worse prognosis for this cohort than the cohort in the FOLFOX4 arm in Cunningham et al.^36^

**Table 1:** Baseline patient and tumour characteristics, and summary of object response

	The PRIME study		Cunningham et al. (2009) ^36^
	Panitumumab + FOLFOX4	FOLFOX4	FOLFOX4
Characteristic	(*n* = 468)^a^	(*n* = 467)^a^	(*n* = 362)
Male (%)	66	61	65
Age, years (%)			
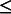 65	60	62	67
65	40	38	33
ECOG performance status (%)			
0/1	95	95	93
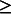 2	5	5	7
Primary tumour type (%)			
Colon	67	69	56
Rectal and other	33	31	44
Number of metastatic sites (%)			
0/1	20	20	45
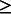 2	80	80	55
Metastatic site (%)			
Liver alone	18	16	33
Prior adjuvant chemotherapy (%)	15	12	27
Prior surgery (%)	91	91	87
Objective response rate (%)	57.9	53.3	54.1

a Results were calculated based on the individual patient-level data obtained from the Project Data Sphere® platform, which have 79% of the subjects randomly selected from the original trial dataset in each arm.

The odds ratio (OR) for the objective response for panitumumab + FOLFOX4 versus FOLFOX4 alone in the PRIME study is 1.20 (95% CI: 0.93 to 1.56) based on the IPD obtained from the Project Data Sphere® platform. The OR from the naïve indirect treatment comparison (ITC) without adjusting for the imbalance in the baseline characteristics is 1.17 (95% CI: 0.88 to 1.54). Both results indicate panitumumab + FOLFOX4 is more beneficial than FOLFOX4 alone for the objective response, but not statistically significant.

For this re-analysis, we assume that Table 1 contains all the potential prognostic factors and effect modifiers. Because metastatic site liver alone is highly correlated with number of metastatic sites, we only include number of metastatic sites in all the subsequent analyses. We assume that once adjusting for the following seven covariates: sex, age, ECOG performance status, primary tumour site, number of metastatic sites, prior adjuvant chemotherapy, and prior surgery, there is no unmeasured confounding. To demonstrate the use of QBA, we consider analysing the data for the following scenarios where we assume certain covariate(s) were not reported in Cunningham et al. (2009)^36^:

- Assuming number of metastatic sites is not reported in Cunningham et al.^36^
- Assuming sex is not reported in Cunningham et al.^36^
- Assuming sex and number of metastatic sites are not reported in Cunningham et al.^36^
- Assuming primary site colon and prior adjuvant therapy are not reported in Cunningham et al.^36^

For each of the scenarios listed above, a standard unanchored STC is conducted by firstly fitting a logistic regression to the IPD from the panitumumab + FOLFOX4 arm in the PRIME study adjusting for the observed covariates where appropriate. The average treatment effect for the population in the FOLFOX4 arm in Cunningham et al.^36^ when treating with panitumumab + FOLFOX4 was predicted using the fitted logistic regression from the first step and simulated covariates for the FOLFOX4 arm in Cunningham et al.^36^ using the NORTA algorithm. The predicted effect for Cunningham et al.^36^ FOLFOX4 arm population was based on the natural scale, that is, predicting the probability of having object response for the simulated individuals. The average absolute treatment was obtained by taking the expectation of the predicted probabilities, and then transformed to the log odds scale for the indirect comparison step.

The standard unanchored STC is then followed by deterministic sensitivity analysis to investigate the impact of unmeasured confounding. The mean of the unmeasured covariates was assumed to be some fixed values, varying from the minimum to the maximum possible value for the mean of that covariates. For all the analyses, bootstrap (with 10,000 replications) is used to derive the appropriate standard error for the adjusted OR. All analyses were performed using R software version 4.3. The program code file and a simulated dataset can be found at https://github.com/SRenScharr/QBA-UM-STC. The data used in the case study can be requested for downloading from the Project Data Sphere’s Data Sharing Platform at the following URL: https://data.projectdatasphere.org/.

### 5.1 *Assuming number of metastatic sites is not reported in Cunningham et al.* ***^36^***

Because the number of metastatic sites is identified as a potential confounder but not reported in Cunningham et al.,^36^ the population-adjusted OR when adjusting for the other six observed covariates (OR: 1.18 with 95% CI: 0.96 to 1.44) is confounded by the number of metastatic sites, hence is biased. Although we do not observe the number of metastatic sites in Cunningham et al.,^36^ the deterministic QBA approach allows us to obtain the unbiased OR by assuming a fixed value for the proportion that the number of metastatic sites is 0/1. We assume a range of values for this proportion as we do not know the truth. This sensitivity analysis quantifies how sensitive the conclusion is to the potential unmeasured confounder, which is the number of metastatic sites in this analysis.

**Figure 2:**
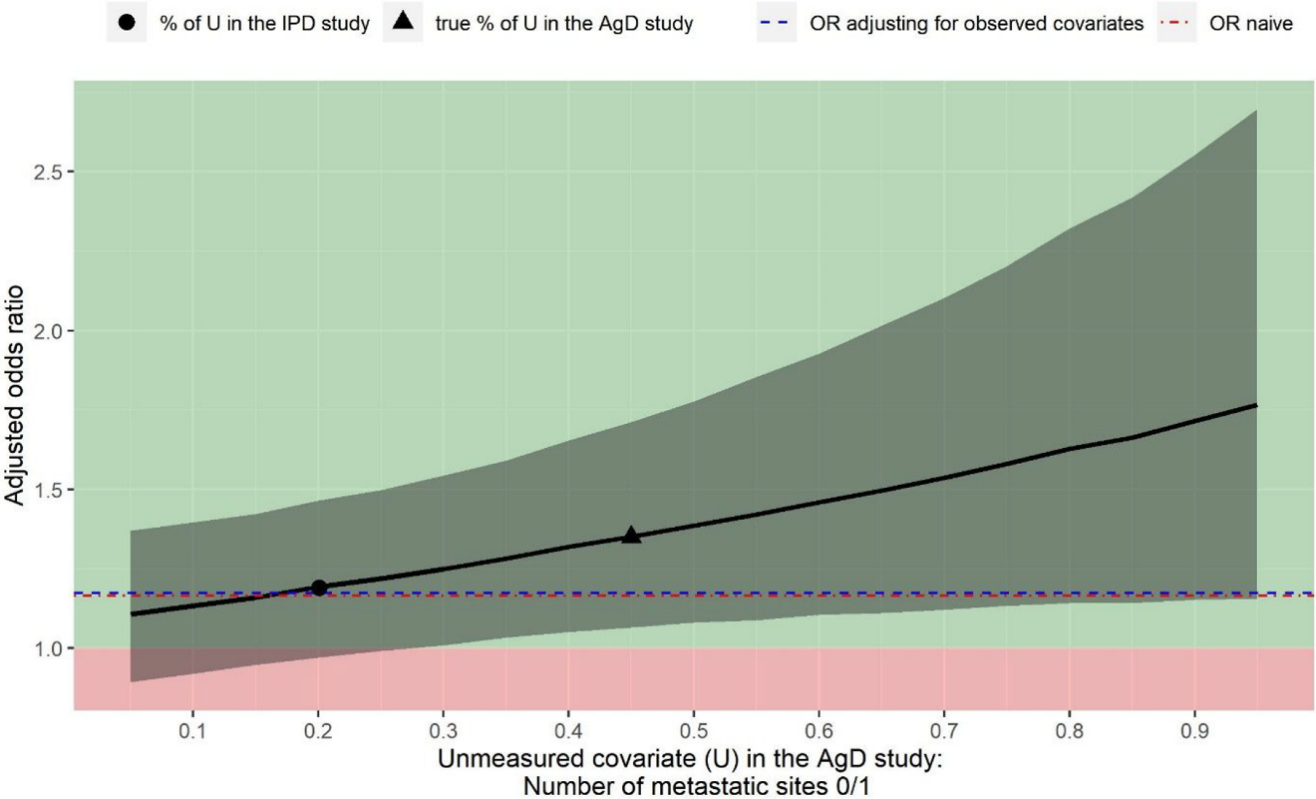
*Sensitivity analysis assuming the number of metastatic sites 0/1 is not reported in Cunningham et al.* ^36^ *Black curve is the estimated treatment effect of panitumumab + FOLFOX4 versus FOLFOX4 alone for the objective response (value above 1 indicating panitumumab + FOLFOX4 is in favour of FOLFOX4 alone). Grey shades indicate the 95% confidence intervals for the estimated odds ratio. Blue dashed line is the odds ratio only adjusting for the observed covariates. Red dot-dash line is the odds ratio derived from a naïve indirect comparison.*

Figure 2 illustrates the output of this sensitivity analysis. The black curve is the OR adjusting for all seven covariates, where the unmeasured confounder (number of metastatic sites) is assumed to vary from 5% to 95%. The adjusted OR increases as the proportion of the number of metastatic sites 0/1 increases. The point estimate suggests that panitumumab + FOLFOX4 would be always more beneficial than FOLFOX4 alone regardless of the value for the proportion of the number of metastatic sites 0/1 observed in Cunningham et al.^36^ If the proportion is greater than 28%, then the treatment effect becomes statistically significant.

Figure 2 also shows the treatment effect when the proportion of the number of metastatic sites 0/1 in Cunningham et al.^36^ is the same as the PRIME study (black circle), which is roughly the same as the OR obtained by only adjusting for the observed covariates. This is as what we expected because when the value for the unmeasured confounder is the same between the two studies, the adjustment including this covariate should not make a difference.

For illustration purposes, we also indicate what the treatment effect would be if the proportion of the number of metastatic sites 0/1 in Cunningham et al.^36^ is equal to the reported value in Cunningham et al.^36^ (black triangular). In this example, unmeasured confounding associated with the number of metastatic sites would not suffice to explain away the treatment effect estimated only adjusting for the observed covariates.

### 5.2 *Assuming sex is not reported in Cunningham et al.* ***^36^***

**Figure 3:**
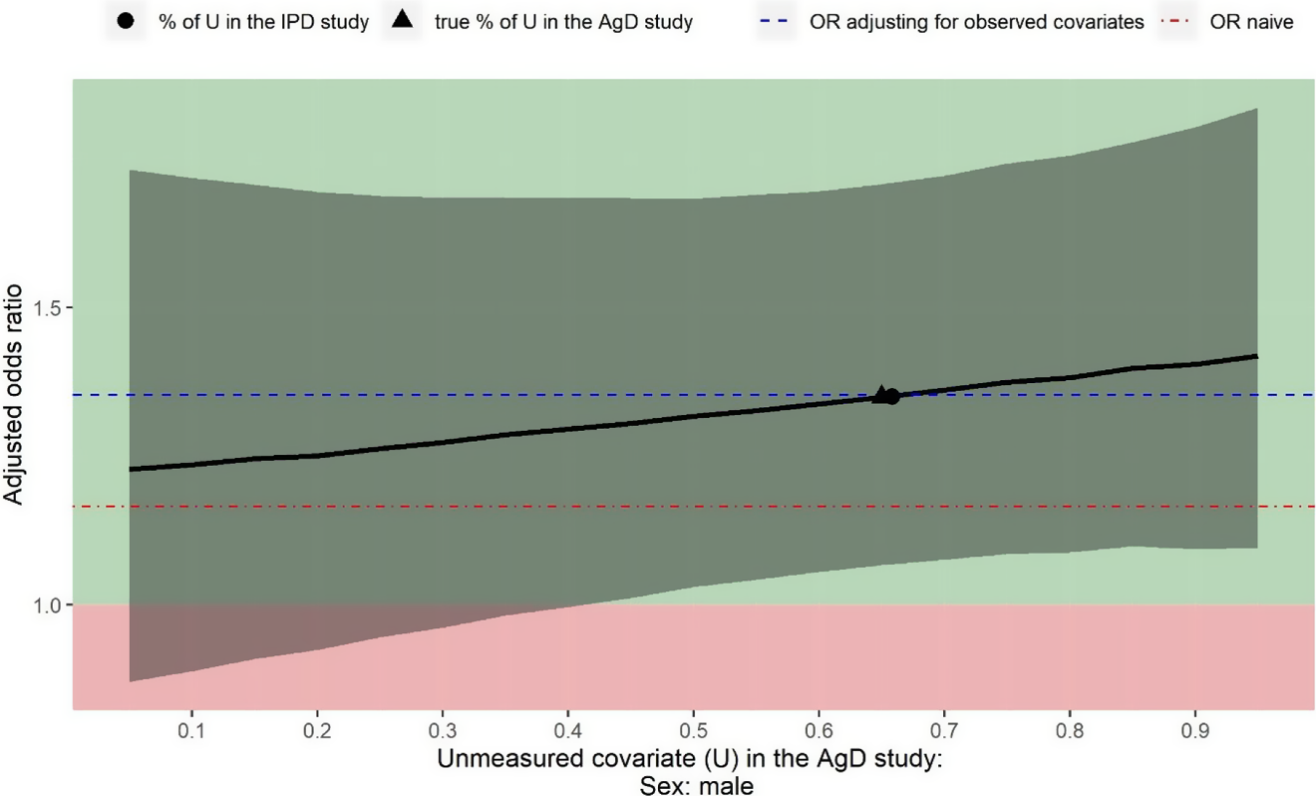
*Sensitivity analysis assuming sex is not reported in Cunningham et al.* ^36^ *Black curve is the estimated treatment effect of panitumumab + FOLFOX4 versus FOLFOX4 alone for the objective response (value above 1 indicating panitumumab + FOLFOX4 is in favour of FOLFOX4 alone). Grey shades indicate the 95% confidence intervals for the estimated odds ratio. Blue dashed line is the odds ratio only adjusting for the observed covariates. Red dot-dash line is the odds ratio derived from a naïve indirect comparison.*

The estimated OR of panitumumab + FOLFOX4 versus FOLFOX4 alone for the objective response when adjusting for six observed covariates is 1.35 (95% CI: 1.07 to 1.71). Figure 3 shows the deterministic sensitivity analysis output for also adjusting for the unmeasured confounder sex, varying values for the proportion of male from 5% to 95%. Regardless of the value for this proportion, the point estimate of the OR is always greater than 1 indicating panitumumab + FOLFOX4 would be more beneficial than FOLFOX4 alone. If there is more than 41% male in Cunningham et al.,^36^ the estimated treatment effect would be statistically significant. This sensitivity analysis demonstrates that unmeasured confounding associated with sex would not suffice to explain away the treatment effect estimated only adjusting for the observed covariates.

### 5.3 *Assuming sex and number of metastatic sites are not reported in Cunningham et al.* ***^36^***

**Figure 4:**
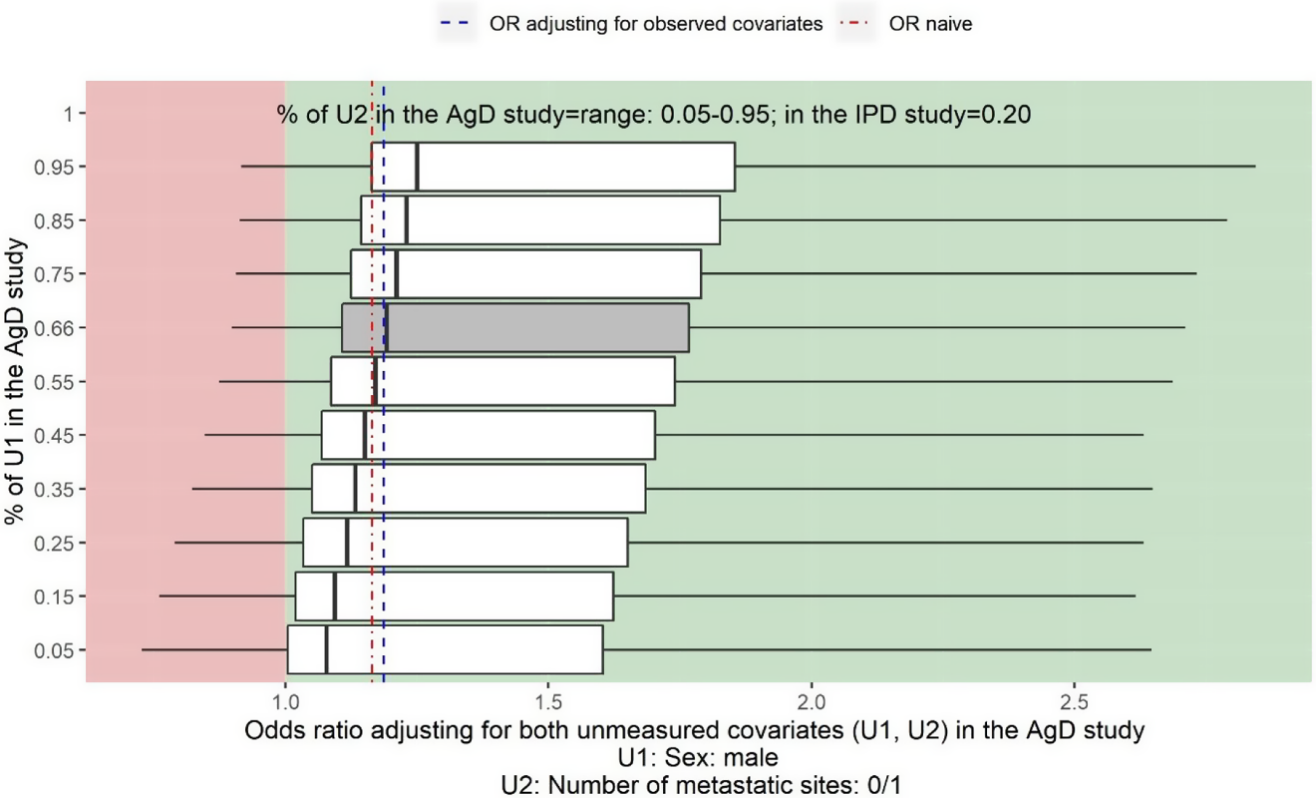
*Sensitivity analysis assuming sex (U1) and number of metastatic sites (U2) are not reported in Cunningham et al.* ^36^ *The vertical line on the left-hand side of the box is the adjusted OR when assuming the proportion of U2 is 5% in Cunningham et al.* ^36^ *for a given proportion of U1 in Cunningham et al.* ^36^ *The vertical line on the right-hand side of the box is the adjusted OR when assuming the proportion of U2 is 95% for a given proportion of U1 in Cunningham et al.* ^36^ *The thick black line inside of the box shows the adjusted OR when the proportion of U2 in Cunningham et al.* ^36^ *is the same as in the PRIME study. The grey box shows the range of the OR when adjusting for U2 assuming the proportion of U1 in Cunningham et al.* ^36^ *is the same as in the PRIME study, 66%. The left-hand side whisker of a box shows the lowest possible value for the lower limit of a 95% confidence interval when varying the proportion of U2 in Cunningham et al.* ^36^ *given a fixed proportion of U1 in Cunningham et al.* ^36^ *The right-hand whisker of a box shows the highest possible value for the upper limit of a 95% confidence interval when varying the proportion U2 in Cunningham et al.* ^36^ *given a fixed proportion of U1 in Cunningham et al.* ^36^

Figure 4 shows the two-way deterministic sensitivity analysis output for adjusting for the unmeasured confounder sex (U1) and the number of metastatic sites 0/1 (U2), varying values for the proportion of male from 5% to 95% and varying values for the proportion of the number of metastatic sites 0/1 from 5% to 95%. The *x*-axis provides the estimate of the OR adjusting for both unmeasured confounders. The *y*-axis indicates the assumed proportion of male in Cunningham et al.^36^

The estimated OR of panitumumab + FOLFOX4 versus FOLFOX4 for the objective response when adjusting for five observed covariates is 1.19 (95% CI: 0.97 to 1.45) (blue dashed line). The estimate for the naïve OR without adjusting for the imbalance in baseline covariates is 1.17 (95% CI: 0.88 to 1.54) (red dot-dash line).

The vertical line on the left-hand side of the box is the adjusted OR when assuming the proportion of U2 is 5% in Cunningham et al.^36^ for a given proportion of U1 in Cunningham et al.^36^ according to the value in the *y*-axis. Similarly, the vertical line on the right-hand side of the box is the adjusted OR when assuming the proportion of U2 is 95% for a given proportion of U1 in Cunningham et al.^36^ The thick black line inside of the box shows the adjusted OR when the proportion of U2 in Cunningham et al.^36^ is the same as in the PRIME study. The grey box shows the range of the OR assuming the proportion of U1 in Cunningham et al.^36^ is the same as in the PRIME study, 66%, and varying U2 from 5% to 95%. The left-hand side whisker of a box shows the lowest possible value for the lower limit of a 95% CI when varying the proportion of U2 in Cunningham et al.^36^ given a fixed proportion of U1 in Cunningham et al.^36^ according to the value on the *y*-axis. Similarly, the right-hand whisker of a box shows the highest possible value for the upper limit of a 95% CI when varying the proportion of U2 in Cunningham et al.^36^ given a fixed proportion of U1 in Cunningham et al.^36^

**Figure 5:**
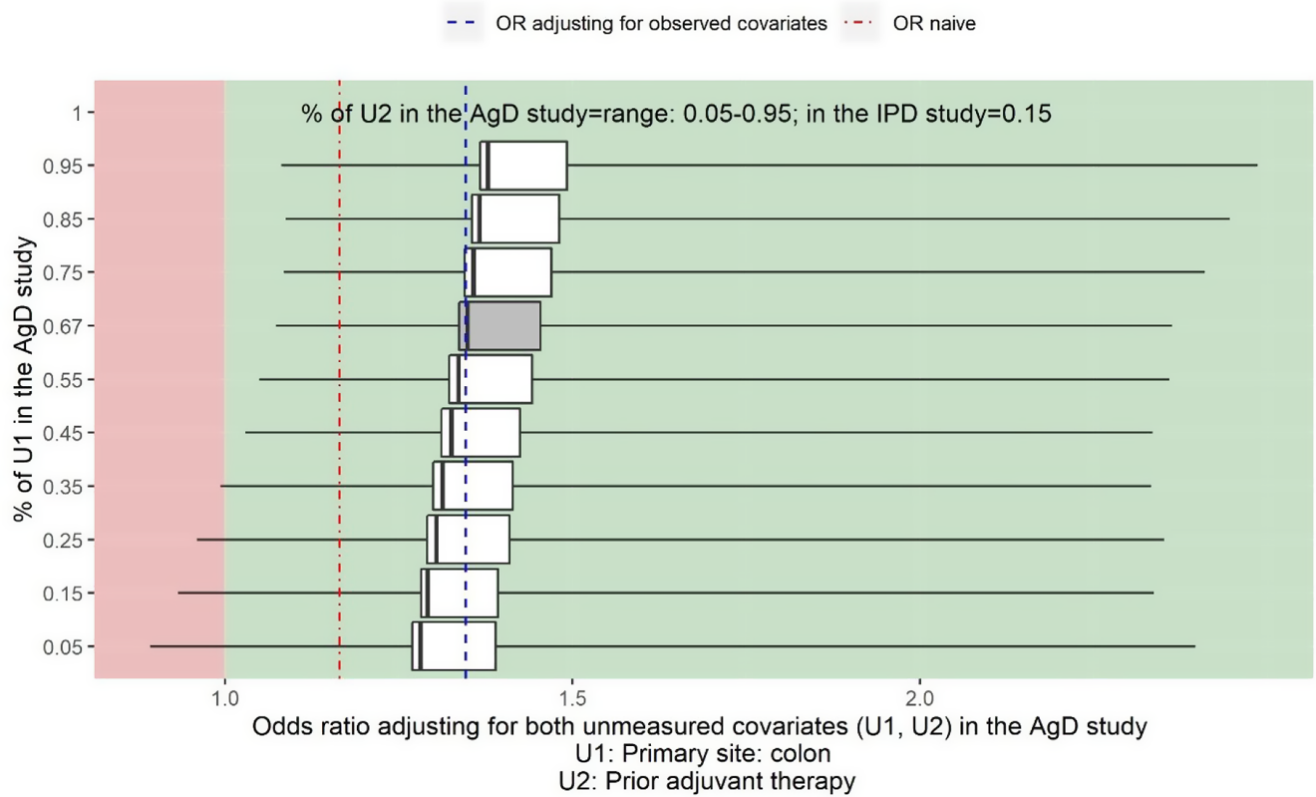
*Sensitivity analysis assuming prior tumour site (U1) and prior adjuvant chemotherapy (U2) are not reported in Cunningham et al.* ^36^ *The vertical line on the left-hand side of the box is the adjusted OR when assuming the proportion of U2 is 5% in Cunningham et al.* ^36^ *for a given proportion of U1 in Cunningham et al.* ^36^ *The vertical line on the right-hand side of the box is the adjusted OR when assuming the proportion of U2 is 95% for a given proportion of U1 in Cunningham et al.* ^36^ *The thick black line inside of the box shows the adjusted OR when the proportion of U2 in Cunningham et al.* ^36^ *is the same as in the PRIME study. The grey box shows the range of the OR when adjusting for U2 assuming the proportion of U1 in Cunningham et al.* ^36^ *is the same as in the PRIME study, 66%. The left-hand side whisker of a box shows the lowest possible value for the lower limit of a 95% confidence interval when varying the proportion of U2 in Cunningham et al.* ^36^ *given a fixed proportion of U1 in Cunningham et al.* ^36^ *The right-hand whisker of a box shows the highest possible value for the upper limit of a 95% confidence interval when varying the proportion U2 in Cunningham et al.* ^36^ *given a fixed proportion of U1 in Cunningham et al.* ^36^

For example, in the top box, the vertical line on the left-hand side of the box is the adjusted OR when assuming the proportion of number of metastatic sites 0/1 is 5% and the proportion of male is 95% in Cunningham et al.^36^ The vertical line on the right-hand side of the box is the adjusted OR when assuming the proportion of number of metastatic sites 0/1 is 95% and the proportion of male is 95% in Cunningham et al.^36^ The thick black line inside of the box shows the adjusted OR when the proportion of metastatic sites 0/1 in Cunningham et al.^36^ is the same as in the PRIME study, 20%, and the proportion of male is 95% in Cunningham et al.^36^

The two-way deterministic sensitivity analysis plot in Figure 4 shows that regardless the value of the unmeasured confounder sex and number of metastatic sites 0/1, the point estimate of the adjusted OR is always greater than 1 indicating panitumumab + FOLFOX4 would be more beneficial than FOLFOX4 alone, however, the treatment effect could not be statistically significant.

### 5.4 *Assuming prior tumour site and prior adjuvant chemotherapy are not reported in Cunningham et al.* ***^36^***

Figure 5 shows the two-way deterministic sensitivity analysis output for adjusting for the unmeasured confounder prior tumour site (U1) and prior adjuvant chemotherapy (U2), varying values for the proportion of prior tumour site colon from 5% to 95% and varying values for the proportion of prior adjuvant chemotherapy from 5% to 95%. The estimated OR of panitumumab + FOLFOX4 versus FOLFOX4 alone for the objective response when adjusting for five observed confounders is 1.35 (95% CI: 1.08 to 1.67) (blue dashed line). The estimate for the naïve OR without adjusting for the imbalance in baseline covariates is 1.17 (95% CI: 0.88 to 1.54) (red dot-dash line).

It shows that regardless the value of the unmeasured confounder prior tumour site and prior adjuvant chemotherapy, the point estimate of the adjusted OR is always greater than 1 indicating panitumumab + FOLFOX4 would be more beneficial than FOLFOX4 alone. When the proportion of prior tumour site colon is greater than 35%, the treatment effect would also be statistically significant regardless of the proportion of prior adjuvant chemotherapy in the study population.

## 6 Discussion

A major limitation of the PAIC methods such as MAIC and STC in an unanchored case is that these adjustment methods rely on the strong assumption that all prognostic factors and effect modifiers are accounted for in the analysis and there is no residual confounding. This assumption is largely considered impossible to meet in practice, especially given the selection of the covariates is often constrained by what baseline characteristics are reported in the comparator study. As a result, the robustness of these methods is often criticised by decision makers and, in turn, may lead to an unfavourable recommendation of the new intervention. Our proposed QBA approach provides a way of conducting both deterministic and probabilistic sensitivity analysis for unmeasured confounder(s) in the unanchored ITC analysis. It allows for formally quantifying the impact of the unmeasured confounder(s) on estimating the relative treatment effect to aid decision-making.

The NICE Decision Support Unit (DSU) Technical Support Document (TSD) 18 advises that sensitivity analyses are performed ‘*to assess how decisions are affected by a range of plausible biases in the effect estimates*’ when performing MAIC/STC.^8^ A recent methodological systematic review found that nearly 50% of the studies did not conduct sensitivity analysis to assess the robustness of PAIC results, and the sensitivity analyses conducted in the literature include adjusting for different set of covariates, applying additional inclusion/exclusion criteria to the IPD study, using different outcome definitions, using different follow-up time.^16^ Although conducting a sensitivity analysis is not new in PAIC methods, there is clear evidence that sensitivity analyses conducted currently are only restricted to observable confounding.

When analysing non-randomised data, observable confounding consists of a fraction of the total error and potential biases due to unmeasured confounder(s), classification errors, and selection bias also need to be addressed to allow for any meaningful interpretation of the results. Greenland discussed basic methods for sensitivity analysis of biases and concluded that ‘*sensitivity analysis is helpful in obtaining a realistic picture of the potential impact of biases*’.^21^ Our proposed QBA approach is inspired by the sensitivity analysis used in observational epidemiology with an aim of providing formal quantitative assessments of bias associated with unmeasured confounding to help decision makers better assess the uncertainty of the ITC results where data were not randomised. Our proposed sensitivity analysis only considers the bias associated with unmeasured covariates. However, there may be other source of heterogeneity between studies such as the study design and conduct, which could bias the treatment effects. We refer the reader to the NICE DSU TSD 18^8^ for some initial suggestions on how to quantify residual systematic error.

We illustrated the use of the QBA approach using a real-world example, and demonstrated how the results of the sensitivity analysis could be presented to aid decision-making. In our case study, we know the unbiased results of the PRIME study as it was an RCT. It is not appropriate to compare the RCT results with the results from the sensitivity analysis because these two results were based on different estimands. The treatment effect derived from the unanchored STC, and the associated sensitivity analyses were the marginal treatment effect in the comparator trial population, which is different from the marginal treatment effect in the RCT population. Analyst needs to determine whether the treatment effect in the comparator trial population is the appropriate measure for the decision problem. The multi-level network meta-regression (ML-NMR) approach for population-adjusted treatment comparisons in anchored cases allows to derive the treatment effect in any target population of interest.^30^ Further research is required for the unanchored case to enable the estimation of the treatment effect in any target population of interest, rather than the comparator study population.

In this article, we only presented sensitivity analyses for unmeasured confounders in the unanchored STC. In theory, the same extension could be applied to other adjustment methods to allow for formal quantitative assessment of uncertainty associated with model assumptions. However, an extension of the MAIC approach would lead to a large reduction of the effective sample size when the overlap in the unmeasured covariates between the studies is small.

Our proposed approach extends the standard unanchored STC by demonstrating how sensitivity analysis can be conducted for unmeasured confounders. Like the original STC approach, our method also relies on the correct specification of the outcome regression model and sufficient population overlap to provide unbiased estimates. The performance of our approach aligns with the properties identified in simulations for the standard unanchored STC that adjusts only for observed covariates.^9^^,^ ^28^^,^ ^37^ Once the sensitivity parameters are specified, the proposed approach effectively reduces to the standard STC method.

We highlight the key factors that most influence the size of the bias here and encourage readers to refer to Ren et al. (2024)^28^ for a more comprehensive discussion on the performance of the unanchored STC approach. The simulation study for unanchored STC shows that sparse data bias together with the degree of covariate overlap between the two studies, are the primary drivers of bias.^28^ When overlap is large or the sample size is big, a large imbalances within covariate strata have little impact on the performance of the unanchored STC method.^28^ Our proposed sensitivity analysis may be biased, particularly when the sample size is small, if the assumed mean values for the unmeasured confounders differ significantly from the observed values in the IPD study.

Two earlier simulation studies on the anchored STC approach assessed the method’s performance when key covariates were missing from the outcome regression model, which represents a form of model misspecification.^9^^,^ ^37^ Both studies concluded that proper covariate selection is critical, and the adjustment method can introduce bias if all necessary covariates are not included in the model. Similarly, our proposed approach would be subject to bias if the outcome regression model is misspecified, resulting in inaccurate predictions and biased estimates of the indirect treatment effect.

In the deterministic sensitivity analysis approach, all possible values for the sensitivity parameters (i.e., the marginal mean of the unmeasured covariates) are treated equally likely, which may not be realistic in practice. An extension of the method is to conduct a probabilistic sensitivity analysis, by constructing a distribution for the sensitivity parameters to reflect the uncertainty for these parameters and obtain the overall results by averaging over this distribution. The area on how to obtain such distributions for the marginal means of the unmeasured confounders requires further research. We provide some initial suggestions here.

The distribution could be elicited using expert’s opinion. It may be easier to elicit a distribution for each 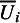 separately. However, this would ignore the correlation structure between the uncertain quantities 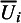 . The method for eliciting a multivariate distribution can be found in Daneshkhah and Oakley.^38^ The distribution could also be estimated using relevant external data via a regression modelling approach based on the observed data. This is to firstly fit a regression model with the unmeasured covariates as outcome and observed covariates as predictors using IPD from the company study or other relevant observational studies that has both the observed and unmeasured covariates of interest reported. The predictions can then be generated by using the observed covariates from the comparator study as predictors. The marginal mean of unmeasured covariates can be predicted separately using a single-output method or jointly using a multi-output regression method that considers the dependency between multiple outcomes.^39^ In addition, machine learning methods could also be used to obtain accurate predictions for the unobserved covariates.^40^

When making funding decisions where the clinical evidence is based on single-arm trials, we face the challenge of implausible model assumptions with the existing analysis methods. Currently, the robustness of the results with regard to model assumptions is often only qualitatively discussed. When analysing data collected without randomisation, it is important to quantitatively assess the uncertainty associated with model assumptions if the intention is to make a causal statement. Our proposed QBA approach using sensitivity analysis relies on the important covariates being measured in the company’s own trial. If any important covariates were not measured in both studies, it would limit what the sensitivity analysis could achieve. We believe when conducting a clinical trial, it is often the case that the important covariates were collected but may not be reported in a publication. The QBA approach makes an attempt to explore if the unmeasured confounder(s) is influential when estimating the treatment effect and, in turn, makes the ITC results more credible.

Finally, we provide guidance on interpreting sensitivity analysis results and practical recommendations for integrating these findings into the decision-making process. Recognising that certain important covariates may not be measured/reported and that their true values remain uncertain, our proposed sensitivity analysis quantifies the impact of unmeasured confounders by quantifying how the results vary with a range of possible mean values for unmeasured confounders. This approach allows for a comprehensive assessment of both the point estimate and its associated uncertainty in the ITC results.

In the case study, the unmeasured confounders did not significantly influence the ITC estimates. All sensitivity analyses indicated a beneficial treatment effect for the new intervention, with some scenarios demonstrating statistical significance depending on the assumed values of the unmeasured confounders.

These sensitivity analysis results can be incorporated into scenario analyses within economic models, explicitly assessing their impact on the incremental cost-effectiveness ratio (ICER). If all scenarios yield ICERs below the threshold, this indicates the robustness of the results, reassuring decision-makers that unmeasured confounding is not a concern in the unanchored analysis. Conversely, if all scenarios produce ICERs above the threshold, this suggests that the treatment is not cost-effective, irrespective of the values of the unmeasured confounders. In cases where decision-making is sensitive to the assumed values of the unmeasured confounders, it is crucial to focus on identifying the most plausible range for these values. This ensures that decisions are well-informed and grounded in realistic assumptions.

## Supporting information

Ren et al. supplementary material Ren et al. supplementary material

## Data Availability

The data that support the findings of this study are available from Project Data Sphere at https://www.projectdatasphere.org/. The program code file and a simulated dataset can be found at https://github.com/SRenScharr/QBA-UM-STC.

## Supplementary material

To view supplementary material for this article, please visit http://doi.org/10.1017/rsm.2025.13.
