# Supplementary material for: Quantitative bias analysis for unmeasured confounding in unanchored population-adjusted indirect comparisons
Source: Res Synth Methods. 2025 Mar 24;16(3):509–27. doi: 10.1017/rsm.2025.13 (PMC12527536; doi:10.1017/rsm.2025.13)
Supplement: Ren et al. supplementary material [file S1759287925000134sup001.pdf]

Appendix 1: The NORTA algorithm

The NORTA algorithm also is known as a Gaussian copula method.[30-32] It is a very flexible approach to simulate multivariate, non-normal data by firstly sampling from a multivariate normal distribution and then transforming the sampled multivariate normal variables into variables with other marginal distributions. To simulate a random vector $\boldsymbol{X} = (X_1, \ldots, X_k)$ with the following properties

- $X_i \sim F_{X_i}$, $i = 1, \ldots, k$ and F_{X_i} is the cumulative distribution function (CDF) for X_i; and
- $Corr(\boldsymbol{X}) = \Sigma_X$,

the NORTA algorithm proceeds as follows:

1. Simulate from $\boldsymbol{Z} = (Z_1, \ldots, Z_k)$, where Z_i follows a multivariate normal distribution with mean 0 and correlation matrix Σ_Z, $i = 1, \ldots, k$, that is $Z_i \sim MVN(0, \Sigma_Z)$.
2. Apply the probability integral transformation to sampled Z_i, such that $V_i = \Phi(Z_i)$, where $\Phi(.)$ is the standard normal CDF and V_i follows a standard uniform distribution, $V_i \sim U[0,1]$.
3. $\boldsymbol{X}$ is obtained based on the marginal distribution of X_i using $X_i = F_{X_i}^{-1}(V_i)$, where $F_{X_i}^{-1}(.)$ is the inverse CDF of X_i.

$$\begin{pmatrix} Z_1 \\ \vdots \\ Z_i \\ \vdots \\ Z_k \end{pmatrix} \xrightarrow[X_i = F_{X_i}^{-1}(\Phi(Z_i))]{} \begin{pmatrix} X_1 \\ \vdots \\ X_i \\ \vdots \\ X_k \end{pmatrix}$$
